# Whole mitogenome sequencing uncovers a relation between mitochondrial heteroplasmy and leprosy severity

**DOI:** 10.1186/s40246-023-00555-8

**Published:** 2023-12-08

**Authors:** Felipe Gouvea de Souza, Moisés Batista da Silva, Gilderlanio S. de Araújo, Caio S. Silva, Andrey Henrique Gama Pinheiro, Miguel Ángel Cáceres-Durán, Mayara Natália Santana-da-Silva, Pablo Pinto, Angélica Rita Gobbo, Patrícia Fagundes da Costa, Claudio Guedes Salgado, Ândrea Ribeiro-dos-Santos, Giovanna C. Cavalcante

**Affiliations:** 1 Laboratório de Genética Humana e Médica, Instituto de Ciências Biológicas, Universidade Federal do Pará, Belém, PA 66075-110 Brazil; 2 Laboratório de Dermato-Imunologia, Instituto de Ciências Biológicas, Universidade Federal do Pará, Marituba, PA 67105-290 Brazil

**Keywords:** Leprosy, mtDNA, Haplogroups, *Mycobacterium leprae*, Mitogenome

## Abstract

**Background:**

In recent years, the mitochondria/immune system interaction has been proposed, so that variants of mitochondrial genome and levels of heteroplasmy might deregulate important metabolic processes in fighting infections, such as leprosy.

**Methods:**

We sequenced the whole mitochondrial genome to investigate variants and heteroplasmy levels, considering patients with different clinical forms of leprosy and household contacts. After sequencing, a specific pipeline was used for preparation and bioinformatics analysis to select heteroplasmic variants.

**Results:**

We found 116 variants in at least two of the subtypes of the case group (Borderline Tuberculoid, Borderline Lepromatous, Lepromatous), suggesting a possible clinical significance to these variants. Notably, 15 variants were exclusively found in these three clinical forms, of which five variants stand out for being missense (m.3791T > C in *MT-ND1*, m.5317C > A in *MT-ND2*, m.8545G > A in *MT-ATP8*, m.9044T > C in *MT-ATP6* and m.15837T > C in *MT-CYB)*. In addition, we found 26 variants shared only by leprosy poles, of which two are characterized as missense (m.4248T > C in *MT-ND1* and m.8027G > A in *MT-CO2*).

**Conclusion:**

We found a significant number of variants and heteroplasmy levels in the leprosy patients from our cohort, as well as six genes that may influence leprosy susceptibility, suggesting for the first time that the mitogenome might be involved with the leprosy process, distinction of clinical forms and severity. Thus, future studies are needed to help understand the genetic consequences of these variants.

## Background

Mitochondria are cytoplasmic organelles that participate in several processes in cellular functioning in humans, including different types of cell death, control of calcium levels, regulation of the immune system, metabolic cell signaling and generation of cellular energy in the form of Adenosine Triphosphate (ATP) by tricarboxylic acid (TCA) cycle and oxidative phosphorylation (OXPHOS) [1–3].

Given their evolutionary origin, mitochondria have their own genetic material (mtDNA), double-stranded circular molecules located in the mitochondrial matrix and associated with the inner membrane of the organelle [4, 5]. The human mitogenome is 16,569 bp in length, with 37 genes—13 of OXPHOS-associated polypeptides, 22 of transfer RNA (tRNA) and two of ribosomal RNA (rRNA)—in addition to non-coding regions, which include the displacement loop (D-loop) region [6–9].

Mitochondria play an extremely important part in the immune system, such as proliferation in the energy supply for the synthesis of signaling and effector molecules, as well as acting directly on signaling pathways for the activation of these cells through intermediary molecules [10]. These include mtDNA, which might act in the pathogenesis process by *Mycobacterium* genus [11], and mitochondrial reactive oxygen species (mtROS), which play a central role in the process of NLRP3 inflammasome regulation and activity. This molecular complex is crucial in the process of releasing proinflammatory oxytocins, such as IL-1b and IL-18 [12, 13].

Importantly, different cells have a variable number of mtDNA copies that can lead to a state called mitochondrial heteroplasmy, which can be characterized as the presence of two or more mtDNA variants in varying proportions within individual organisms [8, 14, 15]. Heteroplasmy is a normal part of healthy human biology, but it is also relevant in disease processes, with the level of heteroplasmy being crucial for the expression of specific pathological phenotypes [2, 8, 15]. In addition, given the importance of mitochondria to the immune system, the accumulation of mutations can lead to mitochondrial dysfunction, which in turn might be responsible for cellular dysregulation, leading to the development and aggravation of multiple infectious diseases [16, 17] such as leprosy.

Leprosy, or Hansen’s disease, is a chronic granulomatous bacterial infection that primarily affects skin and peripheral nerves. The etiological agent is the obligate intracellular bacteria *Mycobacterium leprae*, which produces a broad spectrum of the illness, while diffuse lepromatous leprosy may also be caused by *Mycobacterium lepromatosis*, a new species described in 2008 [18]. Regardless, host factors that regulate susceptibility to the diverse clinical forms of the disease are important, but largely unknown [19, 20]. Leprosy remains a serious public health problem in various parts of the world and, in 2021, more than 140,000 new cases were reported globally, a higher number compared to 127,396 cases reported in 2020, but still a lower number compared to 202,488 cases reported in 2019. However, these data should be observed with great caution because this decrease is probably due to less detection during the COVID-19 pandemic [21, 22].

Leprosy classification is complex and may include clinical, histopathological, microbiological and immunological features. The Ridley–Jopling system classifies leprosy as a spectral disease: in one extreme, there is the polar tuberculoid form (TT), with a low bacterial load, mainly cell-mediated immunity and minor production of specific antibodies. In the other extreme, there is the polar lepromatous form (LL), in which patients show high bacterial load and respond to infection with elevated production of antibodies, as well as lower or absent *M. leprae*-specific cell-mediated immunity. Between these two polar forms, there is the clinically unstable borderline spectrum: borderline-tuberculoid (BT), borderline-borderline (BB) and borderline-lepromatous (BL), with BB being the most unstable form [23].

The complete genome sequence of the *M. leprae* contains 3,268,210 bp and has an average G + C content of 57.8% [24]. By being able to absorb host cell carbon, only about half of the bacillus genome contains functional protein-coding genes [25]. For this reason, *M. leprae* has a dependence on the host’s energy production and nutritional products, resulting in parasitic life adaptation, undoubtedly involving the main function of the mitochondrion, due to the cell signaling pathways in which this organelle participates and connects its metabolism to meet their nutrient demands [26, 27].

Notably, mitochondria have important functions in the regulation of novel immune signaling pathways exerting control over redox metabolism, energy flow, apoptosis, xenophagy and activating inflammasomes. For instance, it has already been shown that leprosy patients have a differential expression of non-coding RNAs such as piRNAs compared to clinically healthy people in the clinical form, as well as in the clinical spectrum of the disease. One of the differential expressions is related to the activation of anti-apoptotic pathways, evidence of the pathogen's interference in the host's mitochondria [28]. In addition, these organelles directly influence intracellular pathogens that attempt to invade their space; inhibition of mitochondrial energy metabolism likely emerges as a novel and overlooked mechanism developed by *M. leprae* to evade xenophagy and the host immune response [25].

In this scenario, mutations that affect mitochondrial functions might influence the host response to this infection, leading to multiple possibilities in leprosy development and outcome. These mutations might be present in the host's mitochondrial genome. Therefore, we sequenced the whole mitochondrial genome to investigate variants and their heteroplasmy levels in the context of leprosy. To the best of our knowledge, this is the first study to perform such genomic approach regarding infection by *M. leprae*.

## Methods

### Sampling

Blood samples were obtained from patients affected by leprosy (*n* = 33, case group) and healthy household contacts with leprosy patients (*n* = 37, control group), all residents of Pará state, Brazil. The case group was composed of borderline lepromatous (BL) leprosy (*n* = 12), lepromatous (LL) (*n* = 11) and borderline tuberculoid (BT) leprosy (*n* = 10). This study adhered to the Declaration of Helsinki and was approved by the Ethics Committee of Institute of Health Sciences at the Federal University of Pará (CEP-ICS/UFPA n. 197/07), and all participants read and signed an informed consent form.

As inclusion and exclusion criteria, the samples from case group were selected from patients affected by leprosy who had a positive clinical and laboratory diagnosis. The samples from control group were selected from healthy household contacts who had a negative clinical and laboratory diagnosis. All participants were recruited at Dr. Marcello Candia Reference Unit in Sanitary Dermatology of the State of Pará (URE) located in Marituba, Pará, Brazil.

### Clinical and laboratory diagnosis

The diagnosis of leprosy was conducted with the well-accepted clinical signs and symptoms based on the Ridley–Jopling classification, including detection of hypopigmented or infiltrated skin lesions with loss of sensation assessed with standard graded Semmes–Weinstein monofilaments and the palpation of peripheral nerves to identify characteristic pain associated with inflammation or swelling, as previously described [29, 30].

To establish laboratory parameters, antibody titers of three antigens (NDO-BSA, LID-1 and NDO-LID) were evaluated with molecular detection of RLEP by qPCR in leprosy patients and contacting patients [29]. The cutoff values of antibody titers were determined using previously described criteria, and the cutoffs for anti-NDO-BSA and anti-LID-1 were 0.295 and, for anti-NDO-LID, the cutoff was 0.475 [29].

To determine qPCR positivity, a standard curve was prepared from purified *M. leprae* DNA extracted from nude mouse footpads, and then five standard dilution points were included in each plate, considering the samples as positive when the fluorescent signal crossed the automatically calculated threshold line [29]. Amplifications with cycle threshold (Ct) ≤ 45 were considered positive for RLEP [29].

### DNA extraction

DNA was extracted by phenol–chloroform method [31]. Quantification of the extracted material was performed with the NanoDrop 1000 spectrophotometer (Thermo Fisher Scientific, Wilmington, DE, USA).

### Amplification and sequencing

Amplification of mtDNA from the total DNA was performed by conventional PCR with specific primers, as described by Cavalcante et al. [1], to cover the entire mitochondrial genome. To verify the quality of the amplification, the samples were applied to a 1% agarose gel and, later, measured in a Qubit 2.0 fluorometer for the library preparation (Thermo Fisher Scientific). Sequencing of the complete mitochondrial genome was performed using Nextera XT DNA Library Preparation Kit (Illumina Inc., Chicago, IL, USA) to prepare the libraries and MiSeq Reagent Kit V3 (600-cycles) (Illumina) for sequencing on the MiSeq System (Illumina), according to the manufacturer’s instructions. During the preparation of the libraries, High Sensitivity D1000 ScreenTape was used on the Agilent 2200 TapeStation System (Agilent Technologies, Santa Clara, CA, USA) to assess the quality of the genetic material.

### Bioinformatics and statistical analyses

After sequencing, we updated the pipeline for bioinformatics analysis previously described [1]. The paired-end sequencing reads (.fastq files) were trimmed with Trimmomatic v.0.39 [32] to remove leading low quality (LEADING:10), trailing low quality (TRAILING:10) and to scan reads with a 3-base wide sliding window, cutting when the average quality per base drops below 10 (SLIDINGWINDOW:3:10) and those reads with length less than 36nt were discarded. After trimming, *fastq* files were aligned with the human reference mtDNA sequence—revised Cambridge reference sequence (rCRS)—using Burrows-Wheeler Alignment tool (BWA, v.0.7) [33]. SAMtools (v.1.15.1) [34] were used for mapping and sorting sequences, while Picard was used to mark the duplicated reads (v.2.27.5, available at https://github.com/broadinstitute/picard).

After preprocessing the sequences in the aforementioned steps, paired-end.bam files were submitted to mutserve for SNP calling, SNP annotation and heteroplasmy detection (https://mitoverse.readthedocs.io/mutserve/mutserve/). For SNP calling, we performed mutserve for each sample with the following quality parameters: mapping quality = 20, base quality = 20 and alignment quality = 30. SNP annotation was based on the rCRS genome annotation (available at https://github.com/seppinho/mutserve). Mutserve outputs SNPs in.vcf file format that was used as input for inferences of mitochondrial haplogroups by HaploGrep (v2) [35]. To reinforce the reliability of the results in the analyses of the variants, only those that passed rigorous additional filters were considered (filter = PASS; coverage ≥ 545 per variant; presence of heteroplasmy). Then, three databases were used for identification of the found variants based on their position: dbSNP (https://www.ncbi.nlm.nih.gov/snp/) [36], ClinVar (https://www.ncbi.nlm.nih.gov/clinvar/) [37] and gnomAD Browser (https://gnomad.broadinstitute.org/) [38], in addition to literature search. R language [39] was employed for statistical analysis and graph generation with the following packages: ggplot2 [40] and UpSetR [41]. *P*-value < 0.05 was considered as statistically significant.

## Results

### Characterization of the cohort

After processing, the mean sequencing coverage of all samples was 1489×, and two low-quality samples were excluded from the analyses. In the studied cohort, we observed similar average age for both case and control groups, as well as for the case subgroups (Table 1). Interestingly, there were more females than males in the LL subgroup, in comparison with the other two analyzed clinical forms (BL and BT), differing from a previous study by our research group that reported more males with this clinical form [42]. However, in the case group considering all clinical forms, we observed more males than females, corroborating a meta-analysis that investigated leprosy in multiple populations, including Brazil [43].

**Table 1:** Demographic characteristics for case and control groups after processing

Groups	Clinical forms	Sex		Age
		Male (%)	Female (%)	
Case	Total	19 (57.58%)	14 (42.42%)	41.5 ± 15.4
	BT	6 (60%)	4 (40%)	41.5 ± 10.3
	BL	8 (66.67%)	4 (33.33%)	43.8 ± 17.2
	LL	5 (45.45%)	6 (54.55%)	39.2 ± 18.2
Control		18 (48.65%)	19 (51.35%)	40.4 ± 16.6

By analyzing the distribution of mitochondrial macro-haplogroups in our cohort, we found that H2, of European (EUR) ancestry, is the most frequent individual haplogroup in both case (32.35%) and control (21.62%), but the Native American (NAT) haplogroups together account for the largest share in both groups (46.88% in case and 43.24% in control) (Table 2). It is also noteworthy that NAT and African (AFR) ancestries presented more diversity of haplogroups than the others.

**Table 2:** Distribution of macro-haplogroups in case and control groups

Mitochondrial ancestry	Macro-haplogroups	General (%)	Case (%)	Control (%)
Native American	A	10.14	17.65	5.41
	B	11.59	11.76	10.81
	C	18.84	14.71	21.62
	D	4.35	2.94	5.41
	Subtotal	44.93	46.88	43.24
European	H2	27.54	32.35	21.62
	U	1.45	0	2.7
	Subtotal	28.99	34.38	24.32
African	L0	1.45	0	2.7
	L1	4.35	5.88	5.41
	L2	13.04	8.82	16.22
	L3	5.80	5.88	5.41
	Subtotal	24.64	18.75	29.73
Asian	M	1.45	0	2.7
	Subtotal	1.45	0	2.7

**Fig. 1:**
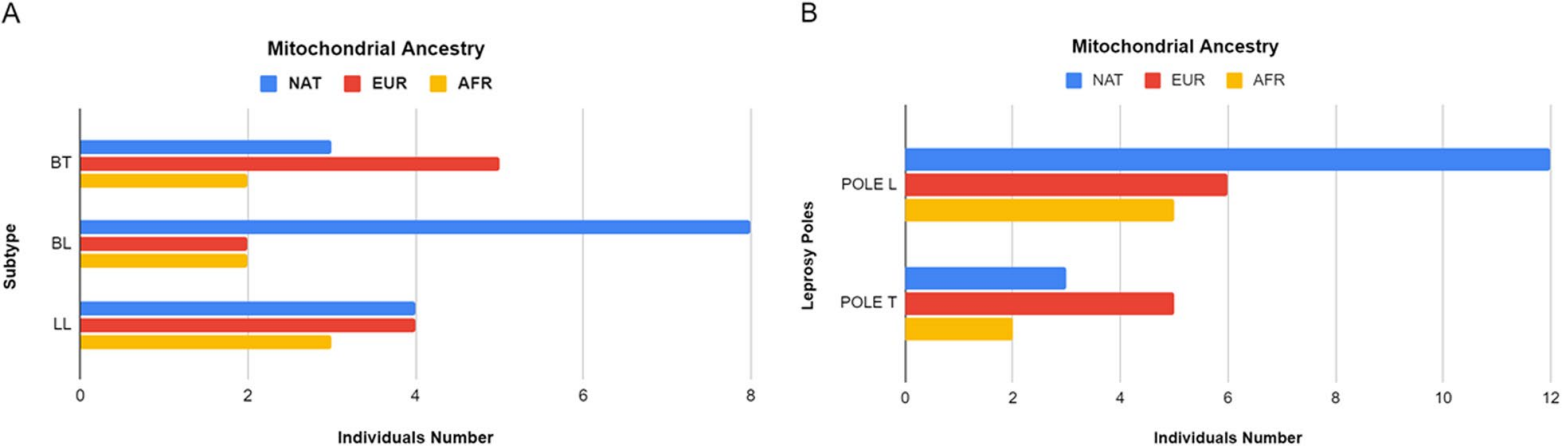
**A** Number of individuals with different leprosy subtypes according to the three mitochondrial ancestries (Native American—NAT, European—EUR and African—AFR) found in the case group. **B** Number of individuals in different leprosy poles (L and T) according to the three mitochondrial ancestries (Native American—NAT, European—EUR and African—AFR) found in the case group

Regarding the leprosy subtypes present in our case group (BT, BL and LL), we found three ancestries based on mitochondrial haplogroups, with different distributions among the subtypes (Fig. 1A). Notably, the Native American ancestry was as frequent as the European ancestry in LL and different in BT, while it was four times more frequent than the other ancestries in BL. Then, an analysis unifying the subtypes into poles was carried out, considering tuberculoid pole (T, with BT, n = 10) and lepromatous pole (L, with BL + LL, n = 23), to assess the mitochondrial ancestry of these individuals considering both poles (Fig. 1B). NAT remained four times more frequent in L than in T, and nearly two times more frequent than EUR and AFR within L pole; in T pole, EUR was nearly two times more frequent than the other two ancestries.

### Distribution of mitochondrial variants

**Fig. 2:**
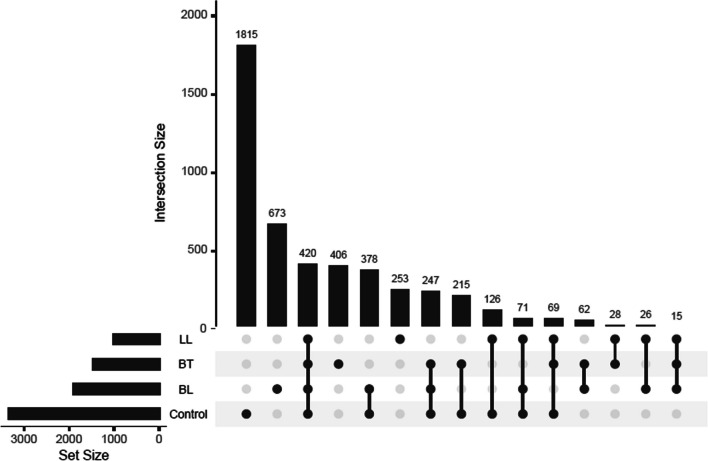
Distribution of found mitochondrial variants in the healthy control group and the leprosy case subgroups (LL, BT and BL). Each dark dot indicates the group with the respective number of variants, and each line represents the intersection between groups. The set size is the overall number of variants

Furthermore, we investigated the distribution of the general mitochondrial heteroplasmic variants present in the study cohort, to assess the variants that were exclusive or shared among the different groups and subgroups, that is, the intersection between groups, as seen in Fig. 2. We found 116 variants to be present in at least two of the subtypes of the case group, but not in the control group. In addition, 1332 variants were found exclusively in one leprosy subtype.

Particularly, 15 mitochondrial variants were exclusively found in all three case subgroups, suggesting they might play a role in the leprosy process: m.3791T > C (*MT-ND1*, no dbSNP identification found), m.5317C > A (*MT-ND2*, no dbSNP identification found), m.8455C > T (*MT-ATP8*, rs1603221490), m.8503T > C (*MT-ATP8*, rs1556423476), m.8545G > A (*MT-ATP8*, rs1603221578), m.9044T > C (*MT-ATP6*, no dbSNP identification found), m.9103T > C (*MT-ATP6*, rs1603222077), m.12879T > C (*MT-ND5*, rs1556424182), m.13512A > G (*MT-ND5*, rs878930809), m.14721G > A (*MT-TE*, rs1603224843), m.14860C > T (*MT-CYB*, no dbSNP identification found), m.14905G > A (*MT-CYB*, rs193302983), m.14941A > G (*MT-CYB*, rs1603224969), m.15019T > C (*MT-CYB*, no dbSNP identification found) and m.15837T > C (*MT-CYB*, no dbSNP identification found). Thus, four are in Complex I genes, five in Complex III, five in Complex V and one in tRNA. Out of these, some variants in the protein-coding genes stand out for being missense and presenting a relevant pathogenicity prediction: m.3791T > C in *MT-ND1* (probably damaging), m.5317C > A in *MT-ND2* (possibly damaging), m.8545G > A in *MT-ATP8* (benign), m.9044T > C in *MT-ATP6* (probably damaging) and m.15837T > C in *MT-CYB* (benign). It is noteworthy that, although most of the variants found in the cohort were detected at low levels of heteroplasmy, recent single-cell analyses have shown that even low-level heteroplasmy (< 5%) can alter transcription levels of nuclear genes involved in ATP synthesis and important cellular processes [44].

**Fig. 3:**
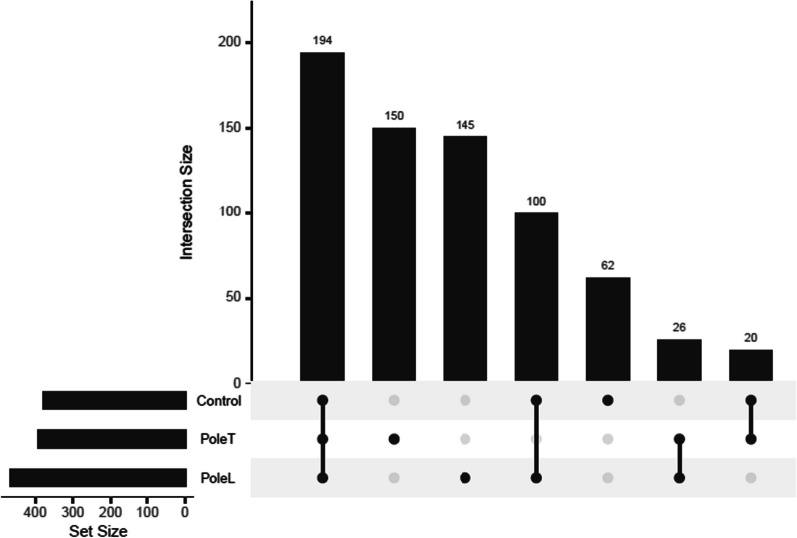
Distribution of found mitochondrial variants in the healthy control group and the leprosy poles (Pole T, and Pole L). Each dark dot indicates the group with the respective number of variants, and each line represents the intersection between groups. The set size is the overall number of variants

Regardless, to analyze the distribution of heteroplasmic mutations in leprosy poles and the intersection between groups, and to exclude variants with low levels of heteroplasmy, we filtered the same variants for those with heteroplasmy levels > 5% and < 95%, to control possible artifacts and false positives (Fig. 3). After this filter, it is possible to notice that 26 variants stand out as shared by both T and L poles.

Of these 26 variants, it is noteworthy that most of them were concentrated in rRNA genes (*MT- RNR2*) and genes encoding OXPHOS complexes, particularly in complexes I (*MT-ND1, MT-ND5*), III (*MT-CYB*) and IV (*MT- CO2* and *MT- CO3*), illustrated in Table 3. This distribution could suggest that at the poles of leprosy there may be a dysregulation of mitochondrial gene expression, as well as in the oxidative environment and energy production, and that these genes may develop roles in disease processes.

**Table 3:** In silico pathogenicity characterization of the variants found only in the leprosy poles

Genes	Mutation	Consequence	dbSNP	ClinVar
*MT-RNR2*	m.1786C > T	Non-coding transcript exon variant	·	·
	m.1752T > A	Non-coding transcript exon variant	·	·
	m.2008G > A	Non-coding transcript exon variant	·	·
	m.1986A > T	Non-coding transcript exon variant	·	·
	m.2001C > T	Non-coding transcript exon variant	·	·
	m.1981G > A	Non-coding transcript exon variant	·	·
	m.1992C > T	Non-coding transcript exon variant	·	·
	m.1773A > G	Non-coding transcript exon variant	·	·
	m.1779A > G	Non-coding transcript exon variant	·	·
	m.1980A > G	Non-coding transcript exon variant	rs1556422588	·
*MT-ND1*	m.4158A > G	Synonymous variant	rs1603219327	Benign
	m.4248T > C	Missense variant	rs9326618	Benign
*MT-ND5*	m.13650C > A	Synonymous variant	·	·
	m.13674T > C	Synonymous variant	rs1603224299	·
	m.12705C > T	Synonymous variant	rs193302956	·
	m.13263A > G	Synonymous variant	rs28359175	·
*MT-CO2*	m.8027G > A	Missense variant	rs1116904	Benign
*MT-CO3*	m.9540T > C	Synonymous variant	rs2248727	·
	m.9950T > C	Synonymous variant	rs3134801	·
	m.9221A > G	Synonymous variant	rs367578507	·
	m.9545A > G	Synonymous variant	rs878853022	Benign
*MT-CYB*	m.14783T > C	Synonymous variant	rs193302982	Likely pathogenic
	m.14905G > A	Synonymous variant	rs193302983	Likely pathogenic
DLOOP1	m.16189T > C	Upstream variant	rs28693675	·
	m.16390G > A	Upstream variant	rs41378955	·
	m.16362T > C	Upstream variant	rs62581341	·

### Analysis of heteroplasmy levels

**Fig. 4:**
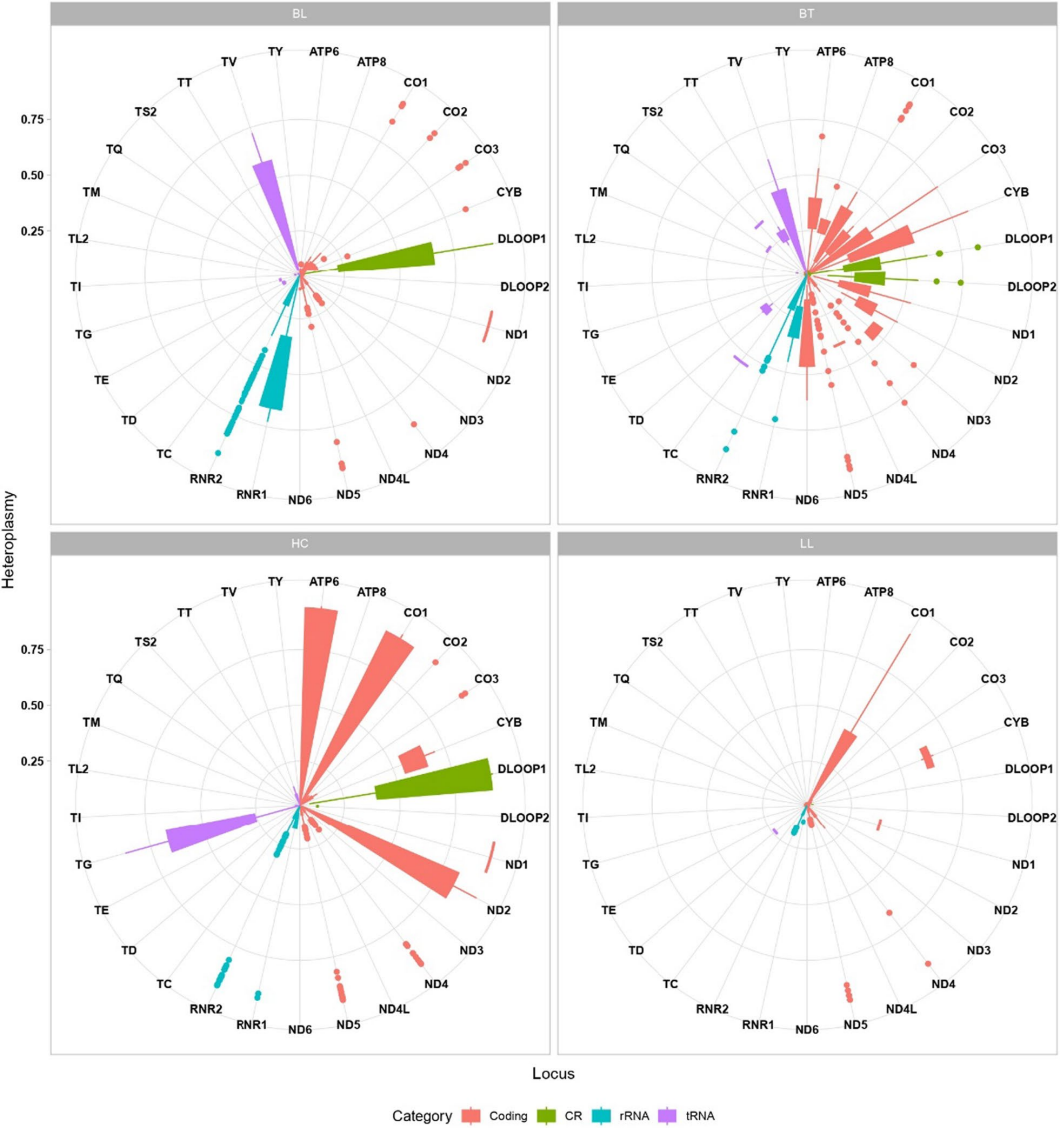
Heteroplasmy levels throughout the mitochondrial genome in healthy controls (HC) and case subgroups (BT, BL and LL). Each region category is color-coded as indicated. The more to the center the boxplot points are, the lower the heteroplasmy rate, as well as the more external the points, the higher the heteroplasmy rate

To investigate mitochondrial heteroplasmy in our cohort, we assessed the levels in which this state was presented in each region of the mitogenome, according to the filtering of > 5% and < 95% (Fig. 4). Interestingly, it seems that tRNAs and control regions (CR) display higher heteroplasmy levels in healthy controls than in leprosy patients, while protein-coding genes have a prominent heteroplasmy variation in the BT subgroup with a widespread pattern in comparison with BL and LL in all OXPHOS protein-coding genes (*MT-ND1*, *MT-ND2*, *MT-ND3*, *MT-ND4*, *MT-ND4L*, *MT-ND5*, *MT-ND6, MT-CYB, MT-CO1, MT-CO2, MT-CO3, MT-ATP6* and *MT-ATP8*).

**Fig. 5:**
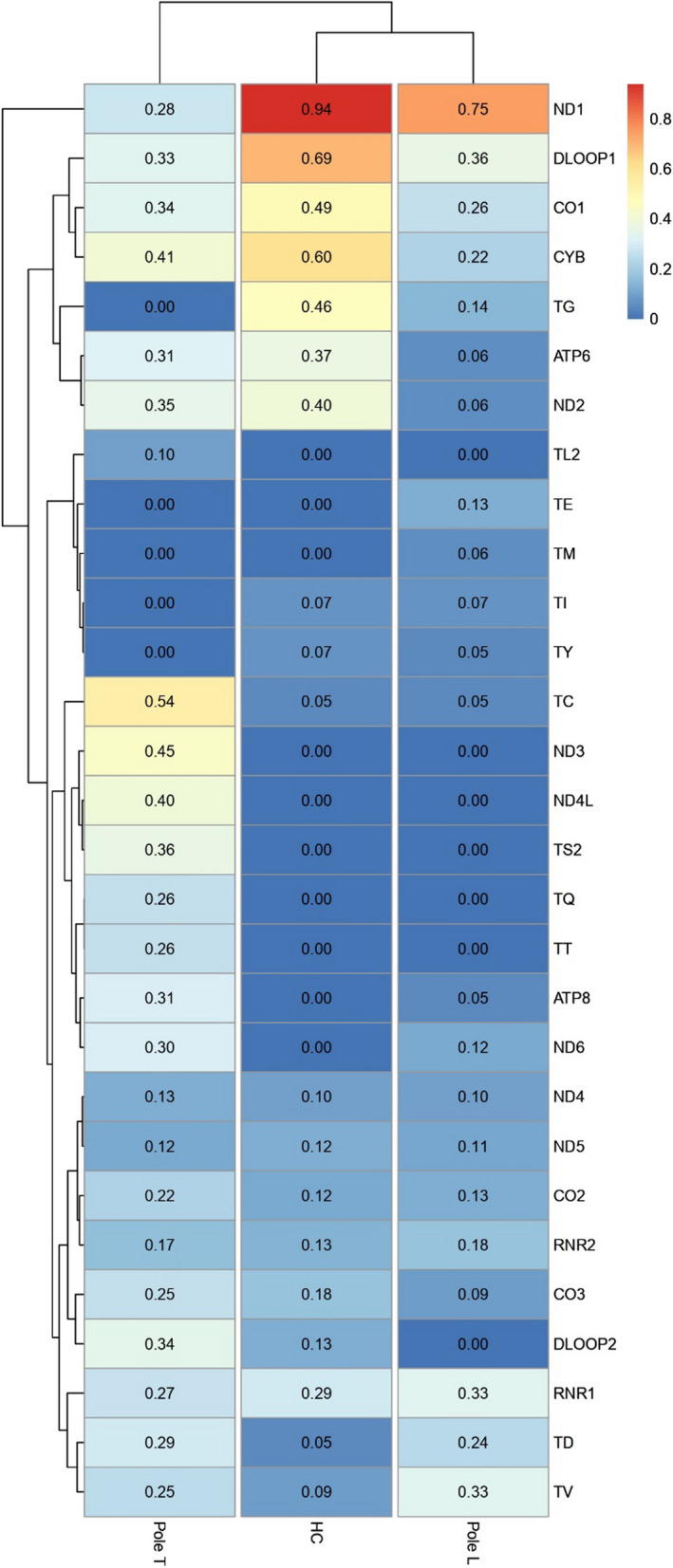
Heatmap displaying heteroplasmy levels of different mitochondrial regions among the healthy control (HC) group and the case subgroups (BT, BL and LL). Similar patterns are clustered together

Moreover, Fig. 5 shows that T pole and L pole present a different pattern of heteroplasmy levels, with L pole being more like healthy controls and T pole being more distant than these states. D-loop presents increased levels of heteroplasmy in T pole when compared to L pole and HC, suggesting that the regulation of mitochondria might be altered in this unstable state.

Strikingly, MT-DLOOP1, *MT-ND1* and *MT-CYB* have a much higher mean of heteroplasmy levels in the control group in comparison with the leprosy clinical forms; not as high, but the heteroplasmy mean levels of *MT-CO1* and *MT-TG* are also elevated when compared to the other groups. In T pole, it should be noted that the MT- DLOOP2, *MT-TC*, *MT-ND3*, *MT-ND4L* and *MT-TS2* also present a higher mean level than the other analyzed groups. Considering that T pole, represented by the BT subtype, is clinically more unstable than the L pole, it is not surprising to see multiple regions with increased heteroplasmy levels in this subgroup, indicating an active inflammatory process with different immune responses [45, 46].

**Fig. 6:**
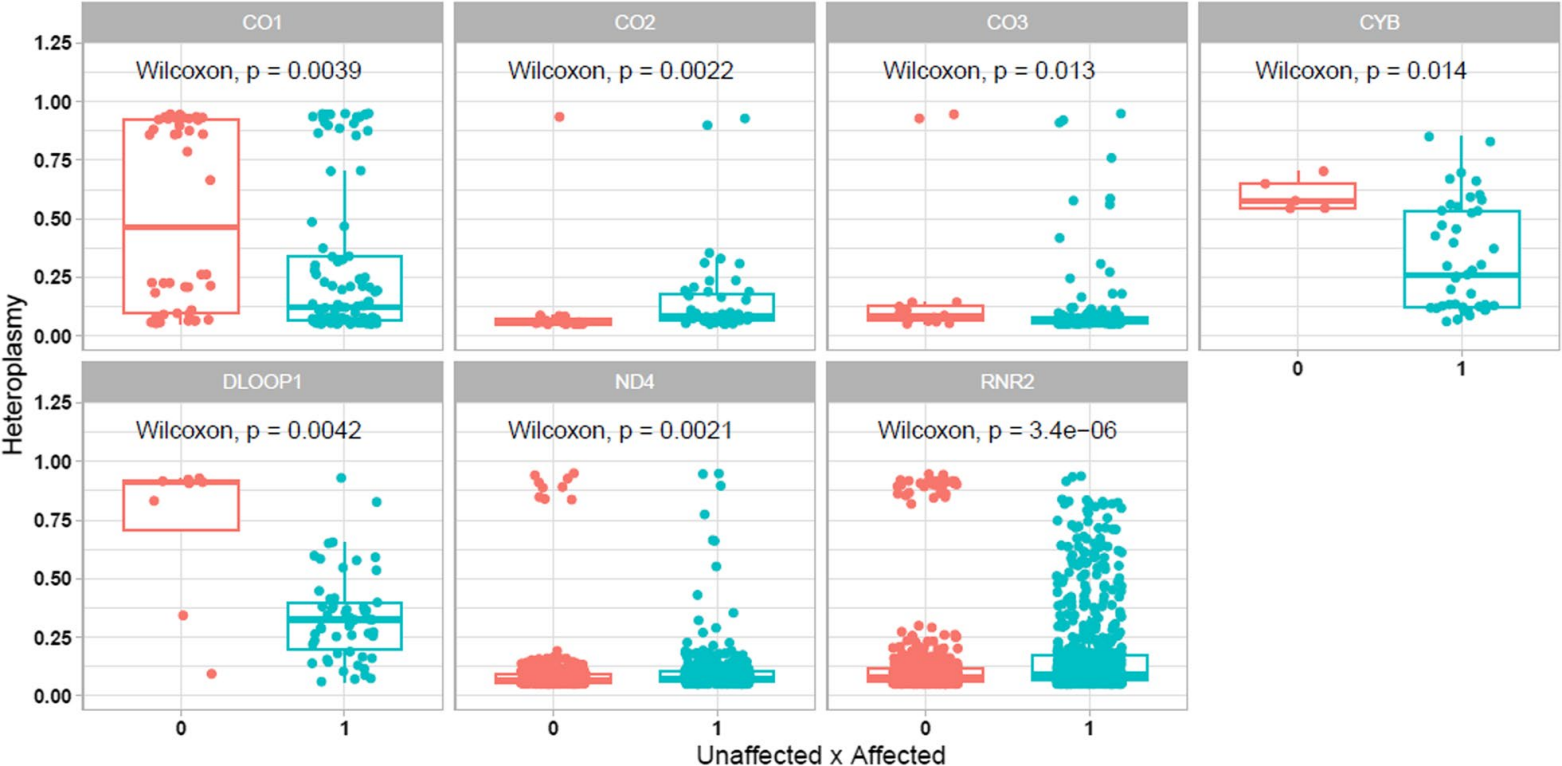
Presence of heteroplasmic variants in control/unaffected (0) and case/affected (1) groups by region of the mitochondrial genome

Furthermore, by analyzing the overall presence of variants and their respective heteroplasmy levels in case and control groups, we found statistical significance in seven regions: one control region, one rRNA and five genes that encode Complexes I, III and IV (Fig. 6). In most of these scenarios, there were more heteroplasmic variants in leprosy patients than in healthy controls, reinforcing the idea that these variants may contribute to the process of susceptibility to leprosy.

**Fig. 7:**
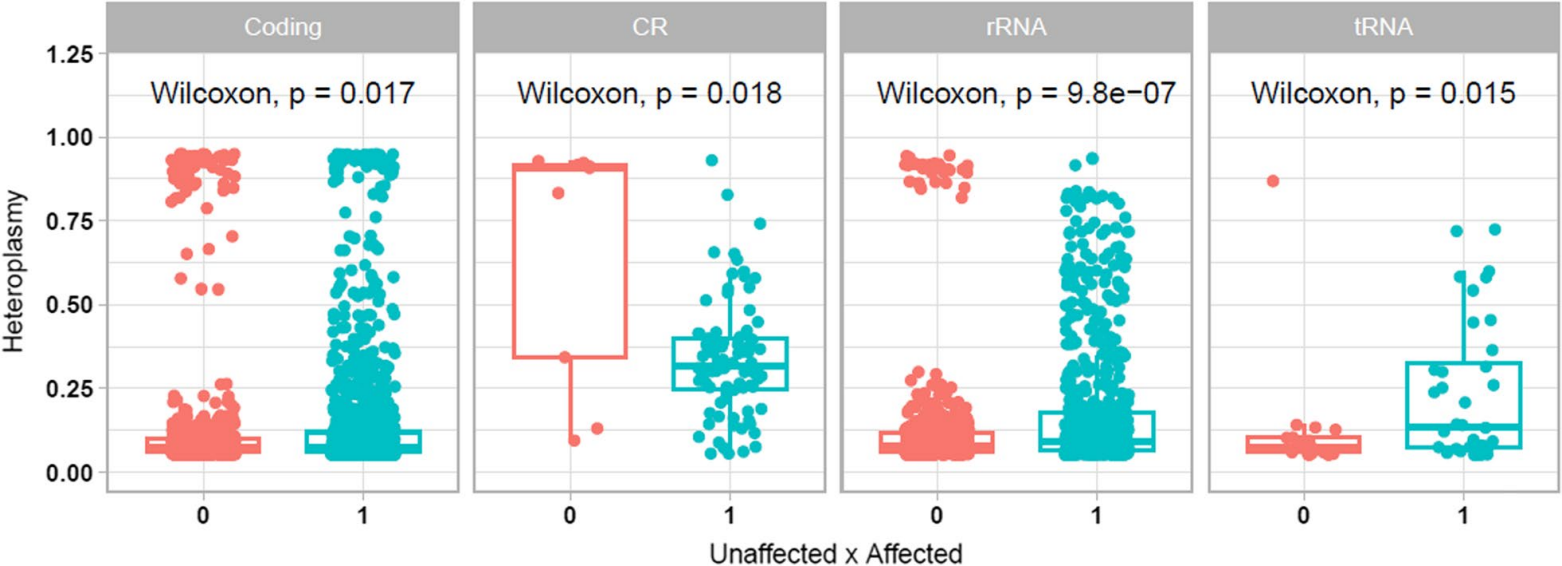
Presence of heteroplasmic variants in control/unaffected (0) and case/affected (1) groups by category of mitochondrial regions

When we considered only the category of mitochondrial regions (coding genes, CR, rRNA and tRNA), we found that all categories still presented statistical significance regarding the presence of heteroplasmic variants (Fig. 7). Notably, there are more variants in the case than in the control, and these variants have diverse heteroplasmy levels, particularly in the rRNA genes. This might reflect the relevant presence of heteroplasmic variants in Complexes I, III, IV, as well as in 16S rRNA, as shown in Fig. 6.

## Discussion

Although leprosy is an important public health problem, it is still neglected and overlooked in research studies, in particular the mechanisms by which the host mitogenome can influence the profile of susceptibility to the disease, especially in the northern region of Brazil [47].

Regarding the results of the analysis of the ancestry profile of the individuals in the cohort, they were expected given the formation process of the Brazilian population and the fact that our cohort is from the North region of the country, in which the Native American ancestry is particularly frequent [48, 49]. Furthermore, the results in Fig. 1 suggest that Native American ancestry could have an influence on the development of different types of leprosy subtypes, particularly BL, upon *M. leprae* infection and reinforced the analysis that mitochondrial ancestry might influence the developed pole.

As for the general distribution of variants illustrated in Fig. 2, no previous studies were found in the global literature with most of these. However, m.15837T > C has been identified in breast nipple aspirate fluid in breast cancer [50]. In addition, we only found a few variants with dbSNP identification, suggesting the remaining variants to be unreported in different databases. Considering three of these variants have been predicted to have a damaging potential, we recommend more studies to clarify their impact in OXPHOS (Complexes I and V) that might affect disease processes such as leprosy.

**Fig. 8:**
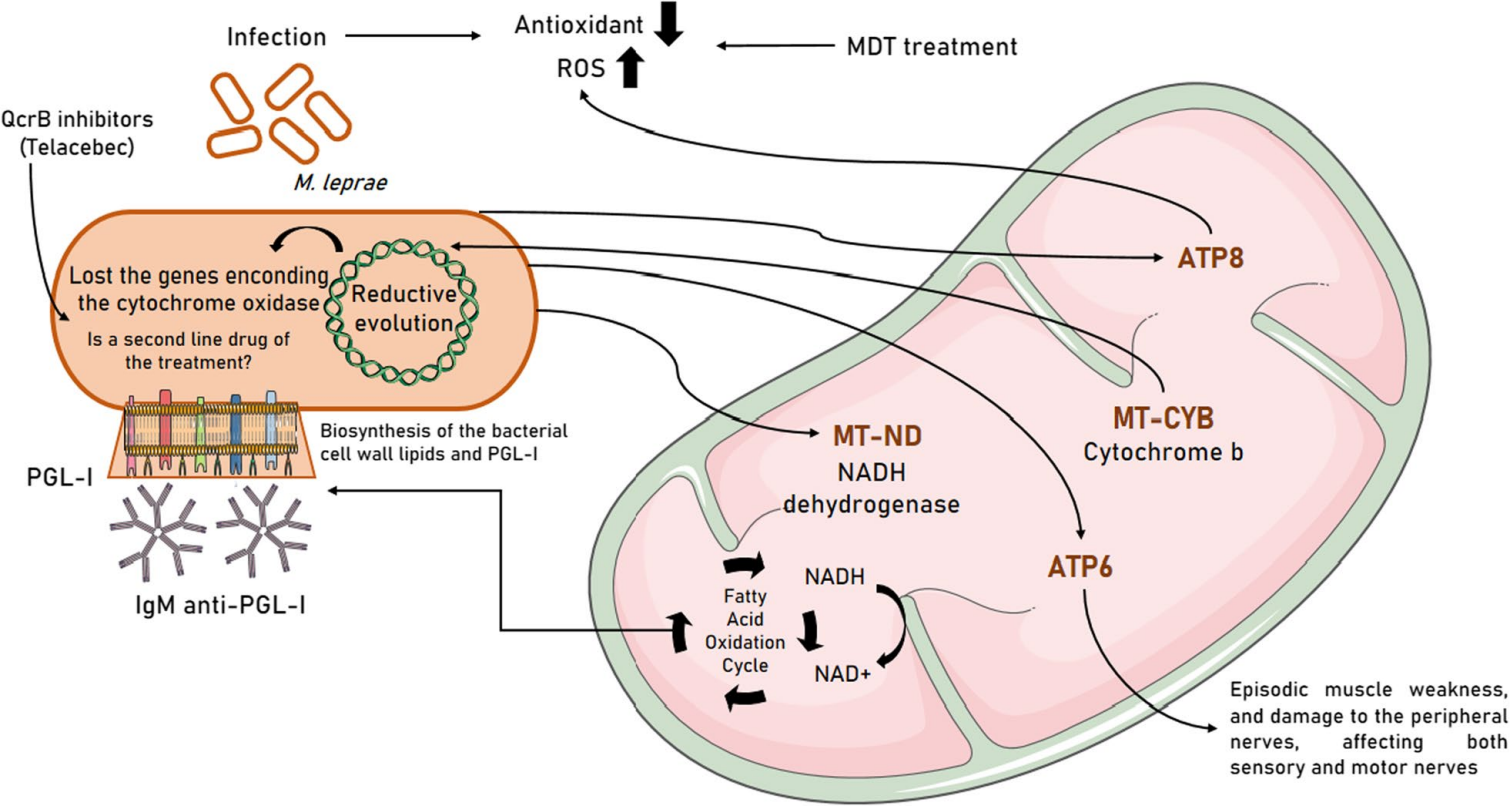
Impact of altered mitochondrial groups of genes in leprosy and their influences on the pathophysiological process of the disease, the viability of the pathogen in the organism, host homeostasis to the immune response and to the therapeutic response

It is important to emphasize that these exclusive mutations are divided into five differential groups: *MT-ND* (*MT-ND1*, *MT-ND2* and *MT-ND5*), *MT-ATP6*, *MT-ATP8*, *MT-CYB* and *MT-TE* genes (Fig. 8). The *MT-ND* genes codify NADH dehydrogenase proteins, composing Complex I. This protein complex transfers the energy generated from the oxidation of NADH to NAD+ and the transfer of protons from the mitochondrial matrix to the intermembrane space through flavin mononucleotide (FMN) and seven to nine iron-sulfur (Fe-S) clusters for the reduction of ubiquinone, the first electron acceptor [51–54].

The NAD + generated by Complex I is used in numerous metabolic reactions by NADH-linked dehydrogenases, including components of the fatty acid oxidation cycle. NADPH is a crucial reductant used in lipid anabolism including synthesis of important components of the mycobacterial cell wall [54] and represents a major electron donor feeding the respiratory chain. Therefore, the NADH molecules generated by 3b-HSD activity could supply, at least in part, electrons to the respiratory chain contributing to *M. leprae* ATP synthesis [54].

The 3b-HSD activity generates the electron donors NADH and NADPH that, respectively, fuel the *M. leprae* respiratory chain and provide reductive power for the biosynthesis of the dominant bacterial cell wall lipids and phenolic glycolipid (PGL)-I [55], the *M. leprae*-specific antigen first reported in 1980 [56], initially tested as a tool for leprosy serodiagnosis [57, 58], but also helping the diagnosis and prediction of relapses [59].

Unfortunately, PGL-I shows low ability to detect true positives, so it is now a well-known biomarker of *M. leprae* exposition and has been used to recognize hidden leprosy cases [60]. Its association with molecular tools or spatial epidemiology is a strong tool to give direction to public policies that lead to an increase in the detection of cases among students and contacts of patients and that, if timely diagnosed, prevent the physical disability of patients, while also breaking the chain of transmission.

Mutations in the *MT-ATP8* gene (a component of Complex V) have been described in reactive oxygen species (ROS) generation [61]. The relationship between *M. leprae* and the increased damage caused by ROS remains unclear, but previous studies have suggested a correlation between bacillary load and oxidative stress [62]. Under chronic inflammatory conditions, ROS reduce activation signals to the T cell and impair the immune response against the pathogen [63]. However, the delicately maintained physiological balance of oxidative stress is changed in favor of ROS from phagocyte and by the treatment (multidrug therapy—MDT), both increasing production of ROS [64, 65] and decreasing antioxidants [63, 66]. Recently, a study verified that elderly patients with leprosy have higher ROS than younger patients [67].

The *MT-ATP6* gene encodes the *ATP6* subunit of mitochondrial ATP synthase (Complex V), which catalyzes the last stage of the electron transport chain (ETC), a key molecular process for the normal axonal function of the central and peripheral nervous system [68, 69]. Mutations in *MT-ATP6* have been linked to episodic muscle weakness [70] and Charcot-Marie-Tooth axonal disease [71]. This disorder damages peripheral nerves and affects both sensory and motor nerves (nerves that trigger an impulse from contraction in muscles) in the arms, hands, legs and feet. Peripheral neuropathies form an integral part of the symptomatology of leprosy and include numbness, painlessness, anesthesia, hypoesthesia and patchy motor deficits, paresthesias (pins and needles), pain (allodynia and dysesthesias), impairment of temperature perception followed by touch and pain; sensory loss, wasting and weakness gradually occur in involved nerve territories and partial involvement [72].

The *MT-CYB* gene codifies a subunit of cytochrome b oxidase (Complex III), involved in oxidative phosphorylation [73], and it has been described as less expressed in leprosy patients when compared to non-leprosy population [74]. The *M. leprae*, like *M. ulcerans*, lost genes encoding cytochrome b oxidase [24] during reductive evolution, making *M. leprae* extremely sensitive to QcrB inhibitors, such as the drug Telacebec [75]. In this context, it should be noted that the established MDT for leprosy was efficient in reducing most cases worldwide, but after 50 years of the same treatment regimen, the increase in resistant cases is the most critical problem [76]. Hence, QcrB inhibitors could represent a new class of bactericidal drugs for leprosy [77], due to their high potency against *M. ulcerans* [78].

The *MT-TE* gene belongs to the set of tRNAs encoded by mtDNA, being important for the biosynthesis of mitochondrial proteins and one of the major causes of disorders in the genome [6, 79, 80]. Pathogenic variants on mitochondrial tRNAs cause a wide range of disease phenotypes, with energy-intensive tissues such as neuromuscular and nervous tissues being particularly vulnerable, with progressive neurological deficits being the most prominent and often the most disabling feature of the disease [79, 81]. Mutations in the *MT-TE* gene have already been associated with the development of diabetes and myopathies, as well as early-onset cataracts, ataxia and progressive paraparesis [81, 82], but there are no reports in the literature about the variant found in our study.

Furthermore, when analyzing the distribution of variants after filtering, 26 variants stand out to be shared in both leprosy poles (Fig. 3). Overall, 16 variants presented identification in dbSNP (Table 3), but only three variants have already been described in the ClinVar as being associated with diseases, such as Leigh syndrome (m.8027G > A) and familial breast cancer (m.14783T > C, m.14905G > A). Two variants were characterized as missense, belonging to *MT-ND1* (m.4248T > C) and *MT-CO2* (m.8027G > A) genes, both being OXPHOS regulators. Interestingly, no previous studies were found in the global literature on most of these variants.

The heteroplasmy analyses suggest that heteroplasmy across the mitochondrial genome can occur differently in each region depending on the affected or unaffected state and the clinical form that leprosy presents (Fig. 4). Therefore, heteroplasmy seems to influence the oxidative environment in the development of this disease. This corroborates previous studies that suggested that the heterogeneity of mtDNA copies might increase during pathophysiological processes and might even be a potential target for therapies of different inflammatory diseases [6, 7, 14]. In leprosy, mitochondrial activity impairment and mtDNA content decrease have been reported, particularly in OXPHOS proteins [25]. This could be related to the observed widespread state of heteroplasmy, especially in the unstable BT clinical form.

The different profile of heteroplasmy between mitochondrial genes at the L, T and HC poles, demonstrated in Fig. 5, suggests that heteroplasmy may influence the type of host immune response to *M. leprae*. The response to leprosy can be classified into two main types: type 1 reaction (T1R) or reverse reaction that occurs mainly in the unstable forms of the disease (BT, BB, BL) and in the TT form and the type 2 reaction (T2R) or erythema leprosy nodosis (ENL) that occurs mainly in BL and LL patients with high bacillary burdens [83, 84].

The different clinical manifestations of leprosy and the histopathology of the two polar forms of leprosy are also determined by the adaptive immune response, the classic paradigm being that the L pole is associated with a Th2 immune response and the T pole with a Th1 immune response [83, 84]. Our findings demonstrate that the heteroplasmic profile of the HC group is more like individuals belonging to the L pole, which suggests that this group tends to be associated with a Th2-type humoral response.

Recent studies have demonstrated that intact mitochondria from human plasma cells harbor immunologically active membrane-associated proteins, including CD270 and programmed cell death ligand 1 (PD-L1) [85]. In addition, intact human plasma mitochondria and immunologically active surface proteins have been associated with upregulation of activated CD4 + T cells and CD8 + T cells and reduced concentrations of pro-inflammatory cytokines [85].

In this perspective, these results suggest that the rate of differential heteroplasmic mutations between genes may be influencing the dysregulation of mitochondrial functions, which may induce a distinct immune response among individuals, dividing them in the clinical forms of leprosy. Importantly, it should be noted that, for many infectious diseases, host genetic factors have long been considered a major contributor to variations in individual susceptibility and immune response [84].

## Conclusion

Here, we reported the whole mitochondrial genome sequencing of leprosy patients and healthy unrelated household controls from a Brazilian population. Importantly, this is likely to be the first study to report a clear association between mitochondrial heteroplasmy and leprosy. This unprecedented approach showed that a higher number of mitochondrial variants and diverse heteroplasmy levels are significantly found in the leprosy patients from our cohort, suggesting for the first time that the mitochondrial genome, and particularly heteroplasmy, may be involved in the inflammatory response observed in the peripheral nerve trunks and in the skin of leprosy cases, as well as in defining the immunological response that determines the clinical polarization of leprosy.

Furthermore, we identified 26 heteroplasmic variants shared between the T and L poles that are present in the *MT-RNR2*, *MT-ND1*, *MT-ND5*, *MT-CYB*, *MT-CO2* and *MT-CO3* genes, suggesting that these genes may be correlated with the susceptibility and severity of leprosy. The infectious process of leprosy has a direct impact on mitochondrial functions, and their dysregulation is objectively involved in the pathophysiological process of the disease, in the viability of the pathogen in the organism, in the host's homeostasis to the immune response and in the therapeutic response. Thus, further clinical studies with larger cohorts with sequencing of other tissues and/or functional studies regarding the found variants must be carried out to expand knowledge about the pathogenesis of *M. leprae* and its possible impacts on the mitochondrial genetic profile of affected individuals and, thus, suggest potential biomarkers for leprosy.

## Data Availability

All raw sequences are deposited at the European Nucleotide Archive (ENA) under the accession number PRJEB59275.
